# Proteomic analysis of JAK2V617F-induced changes identifies potential new combinatorial therapeutic approaches

**DOI:** 10.1038/leu.2017.143

**Published:** 2017-06-16

**Authors:** S Pearson, A J K Williamson, R Blance, T C P Somervaille, S Taylor, N Azadbakht, A D Whetton, A Pierce

**Affiliations:** 1 Stem Cell and Leukaemia Proteomics Laboratory, Manchester Academic Health Science Centre, University of Manchester, Wolfson Molecular Imaging Centre, Manchester, UK; 2 Leukaemia Biology Laboratory, Cancer Research UK Manchester Institute, University of Manchester, Manchester, UK; 3 Stoller Biomarker Discovery Centre, University of Manchester, Manchester, UK

## Abstract

In excess of 90% of patients with polycythaemia vera (PV) express a mutated form of Janus kinase 2 (JAK2), JAK2V617F. Such aberrant proteins offer great potential for the treatment of these diseases; however, inhibitors to JAK2 have had limited success in the clinic in terms of curing the disease. To understand the effects of this oncogene in haematopoietic cells with the aim of improving treatment strategies, we undertook a systematic evaluation of the effects of JAK2V617F expression using proteomics. The effects of JAK2V617F on over 5000 proteins and 2000 nuclear phosphopeptide sites were relatively quantified using either SILAC or eight-channel iTRAQ mass spectrometry. Pathway analysis of the proteins identified as changing indicated disruption to the p53 and MYC signalling pathways. These changes were confirmed using orthogonal approaches. The insight gained from this proteomic analysis led to the formation of hypothesis-driven analysis on inhibitor-mediated effects on primary cells from patients with a JAK2V617F mutation. Simultaneous inhibition of MYC and upregulation of p53 led to the preferential extinction of JAK2V617F-positive CD34+ cells, illustrating a potential therapeutic benefit from combined targeting of p53 and MYC.

## Introduction

Myeloproliferative neoplasms (MPNs) are a heterogenous group of clonal disorders originating within the haematopoietic stem cell compartment. They are characterised by an increased production of myeloid cells with minimal effects on terminal differentiation. They are often associated with a dysregulated protein tyrosine kinase.^1, 2, 3^ The JAK2V617F mutation results in constitutive activation of Janus kinase 2 (JAK2) and is associated with over 90% of patients with polycythaemia vera (PV), and about half of patients presenting with myelofibrosis or essential thrombocythaemia.^4^ Other mutations in JAK2 are found in a minor but significant number of PV cases (<4%) including the JAK2K539L mutation.^5^ Other mutually exclusive mutations are found in calreticulin and myeloproliferative leukaemia virus oncogene.^6, 7, 8^

The advent of tyrosine kinase inhibitors targeting the leukaemogenic tyrosine kinases offered great hope; however, despite their ability to induce durable cytogenetic and molecular responses, they are rarely curative in chronic myeloid leukaemia (CML) and never in other MPNs.^9^ JAK2 inhibitors offer significant benefits in terms of symptom reduction^10, 11^ but few patients display reduced allele burden.^12, 13^ Thus, the development of knowledge of molecular pathogenesis mechanisms for the MPNs offers opportunities to inform potential new treatment strategies.

We have therefore systematically analysed the effects of JAK2V617F by mass spectrometry. The aim was to identify the downstream effectors that may offer opportunity for therapeutic intervention. We demonstrate that many of the JAK2V617F-driven changes in protein expression are regulated by perturbation in transforming growth factor-β (TGFβ), p53 and MYC pathways. Furthermore, by inhibiting MYC and upregulating p53 we are able to preferentially drive the differentiation and death of JAK2V617F-expressing cells from patients with MPN.

## Materials and methods

### Cell lines and peptide/phosphopeptide identification and quantification

Details can be found in Supplementary Methods. In brief, Ba/F3 cells were retrovirally transfected with Epo receptor or Epo receptor plus wild-type JAK2 or mutants K539L and V617F. Epo receptor is required for the JAK2 mutants to transform haematopoietic cells.^14^ For SILAC (stable isotope labelling by/with amino acids in cell culture) labelling, JAK2wt cells were cultured in ‘light’ SILAC media containing ^12^C_6_-Lys and JAK2V617F-expressing cells in ‘heavy’ SILAC media containing ^13^C_6_, ^15^N_2_-Lys for five passages before confirming complete protein labelling by mass spectrometry. SILAC-labelled peptides were analysed on a LTQ Orbitrap Velos (Thermo Fisher, Hemel Hempstead, UK) following analytical separation of the peptides using Acclaim PepMap RSLC C18 Columns. Nuclear proteins were enriched using a kit from Active Motif (La Hulpe, Belgium) and labelled with eight-channel iTRAQ reagent (SCIEX, Warrington, UK), phosphopeptides were enrichment via Ti0_2_ chromatography and labelled peptides analysed on 5600 TripleTOF (SCIEX) as previously described.^15^

### Data analysis and validation

Details are available in Supplementary Methods. In brief, mass spectrometry data from SILAC-labelled samples were processed using MaxQuant (version 1.5.5.1) (false discovery rate set at 0.01) and iTRAQ-labelled samples using ProteinPilot 3 software (SCIEX). All protein and phosphopeptide quantification ratios were checked to ensure they had a normal distribution (Supplementary Figures 2). A protein/phosphopeptide change was defined for each run (Supplementary Table 1) as a ratio outside the range in which 95% of protein/phosphopeptide ratios for the internal replicate were found with a *P*-value of ⩽0.05. Where indicated protein expression was assessed using capillary-based technology of Peggy-Sue (Protein Simple, Abingdon, UK) as previously described^16^ (and in Supplementary Methods). Antibodies used are shown in Supplementary Table 2. Densitometry was performed with ImageJ software (NIH, Bethesda, MD, USA).

### Primary cell assays

Patient samples were obtained from the Manchester Cancer Research Centre Biobank (HTA 30004) and had ethical approval from the National Research Ethics Service committee (14/LO/0489). Experimental details are available in Supplementary Methods. CD34^+^ cells were enriched using CliniMACS (Miltenyi Biotec, Bisley, UK) and colony-forming assays performed in methylcellulose complete media (R&D Systems, Abingdon, UK) supplemented with 2 U/ml erythropoietin at a density of 3000 cells per ml. To assess retention of self-renewal capacity, the resulting colonies at day 7 were replated in methylcellulose and colonies counted at 14 days. CD34^+^ cells were stained using CellTrace Violet Cell Proliferation Kit (Molecular Probes, Thermo Fisher) and then seeded at a density of 1.5 × 10^5^/ml in Iscove’s modified Dulbecco’s medium, 20% (v/v) fetal calf serum, recombinant human interleukin-3 (20 ng/ml), recombinant human stem cell factor (50 ng/ml) and Flt-3 (Fms-related tyrosine kinase 3) ligand (10 ng/ml) (PeproTech, London, UK). On day 0 and following 8 days of culture, cells were harvested and stained with CD34-APC (eBioscience, Thermo Fisher) and appropriate controls using standard protocols. Fluorescence was measured on a LSRFortessa (Becton Dickenson, Oxford, UK) flow cytometer. Results were analysed using FloJo software (Ashland, OR, USA).

## Results

### The effect of expression of JAK2V617F on the proteome

To gain an understanding into the JAK2V617F-driven transformation process we performed a global proteomic screen using SILAC and liquid chromatography/tandem mass spectrometry^17^ on cells expressing wild-type JAK2 and mutated JAK2V617F. We identified 5021 proteins (Supplementary Table 3). Of the proteins quantified with >2 peptides, 140 showed a greater than twofold change, and with >3 peptides, 86 proteins were identified as changing (Supplementary Table 4). These included proteins intrinsically associated with haematopoiesis such as the growth factor TGFβ and the protein phosphatase PTPRC (protein tyrosine phosphatase, receptor type C).

**Figure 1:**
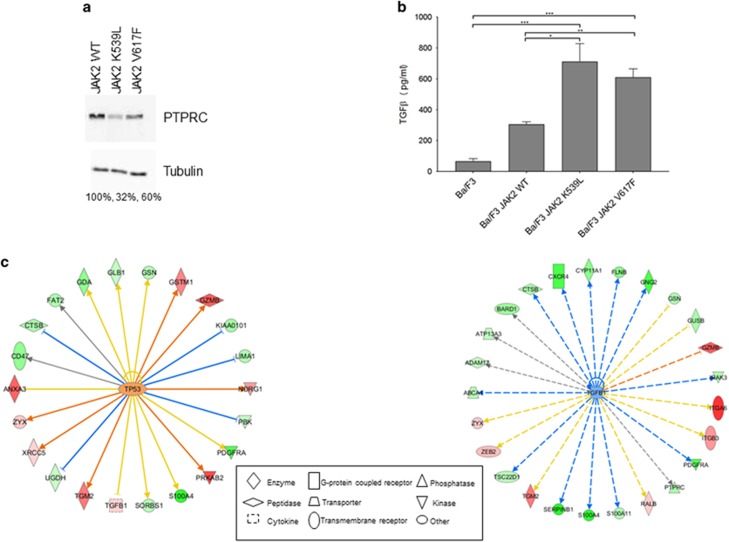
(**a**) Western blot of PTPRC expression in Ba/F3 whole cell lysates (tubulin used as a loading control). Protein expression as a percentage of JAK2WT levels are indicated. (**b**) TGFβ levels were measured in Ba/F3 cell culture supernatants using the Quantikine ELISA kit (R&D Systems). Results are displayed as pg/ml of cell culture supernatant (*n*=3±s.e.m.). The *t*-test results are represented by *P<0.05, **P<0.01, ***P<0.001. Proteins identified as changing (ratio >2-fold with ⩾2 peptides) were subjected to Ingenuity software analysis (**c**) to predict proteins potential responsible for the observed changes. Proteins and lines joining the components are colour coded to represent the changes observed (red and orange upregulated, green and blue downregulated, grey no prediction and yellow a change in the opposite direction to that of the predicted change).

To validate the SILAC data set we assayed PTPRC and TGFβ levels by orthogonal methodologies. PTPRC and TGFβ were chosen because we have previously reported a reduction in PTPRC expression^18^ and upregulation of TGFβ expression^15^ induced by a range of other leukaemogenic oncogenes.^19^ In addition, TGFβ plays an important role in the pathogenesis of CML^20^ and myelofibrosis.^21^ Western blot analysis of PTPRC expression in the presence of activated JAK2 mutants (Figure 1a) demonstrated decreased levels compared with control cells. In MPNs haematopoietic stem cells are thought to secrete factors such as TGFβ that influence the bone marrow niche and haematopoietic cells.^22, 23^ We therefore measured the levels of secreted TGFβ rather than intracellular levels. Figure 1b demonstrates that JAK2V617F and JAK2K539L expression leads to an increase in the secretion of TGFβ.

### Disruption of the TGFβ and p53 pathways

**Figure 2:**
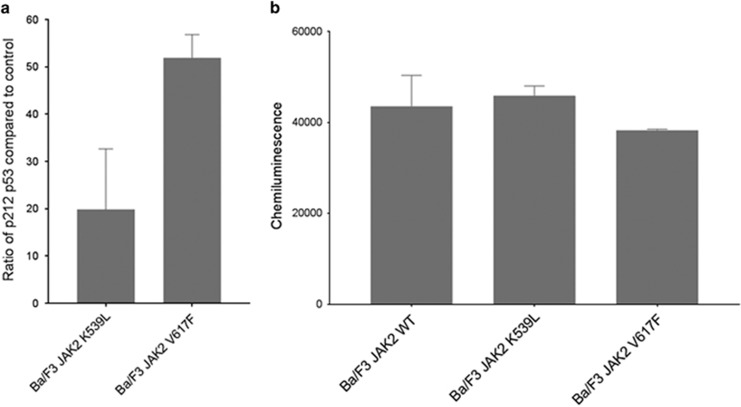
(**a**) A total of 250 fmol of a heavy labelled isotopomer of the p53s212 phosphopeptide was spiked into 100 μg of Ba/F3 cell lysate. Endogenous and heavy labelled peptides were identified and quantified using multiple reaction monitoring and the relative abundance of endogenous to heavy labelled peptide calculated. (**b**) Protein expression of p53 was assessed on Peggy-Sue (Protein Simple). Expression levels were normalised using actin and the quantification illustrated using the chemiluminescent peak area (*n*=3±s.e.m.).

Having validated our SILAC data an *in silico* analysis, using the Ingenuity Pathways Analysis tool, of all the JAK2V617F-induced protein changes predicted a role for TGFβ and p53 (*P-*values of 2.2 × 10^−6^ and 7.4 × 10^−6^ respectively) as major regulators of the observed changes (Figure 1c). There is growing evidence that it is not only the mutational status of p53 that is important in cancer but also its transcriptional activity,^24^ and a role for perturbation of p53 stability has been suggested to play a role in PV progression.^25^ We therefore investigated a key phosphorylation site, serine 212, that has been demonstrated to be critical in the transcriptional activity of p53 in relation to differentiation and self-renewal^26^ rather than apoptosis. Figure 2a shows that there is an increase in the stoichiometry of phosphorylation on serine 212 of p53 in the presence of JAK2K539L and JAK2V617F with no change in p53 protein expression level (Figure 2b). These results suggest that p53 transcriptional activity is reduced^26^ in the JAK2V617F-expressing cells that potentially disrupts the self-renewal/differentiation balance of the HSCs.

### JAK2V167F- and JAK2K539L-induced nuclear proteomic changes

**Figure 3:**
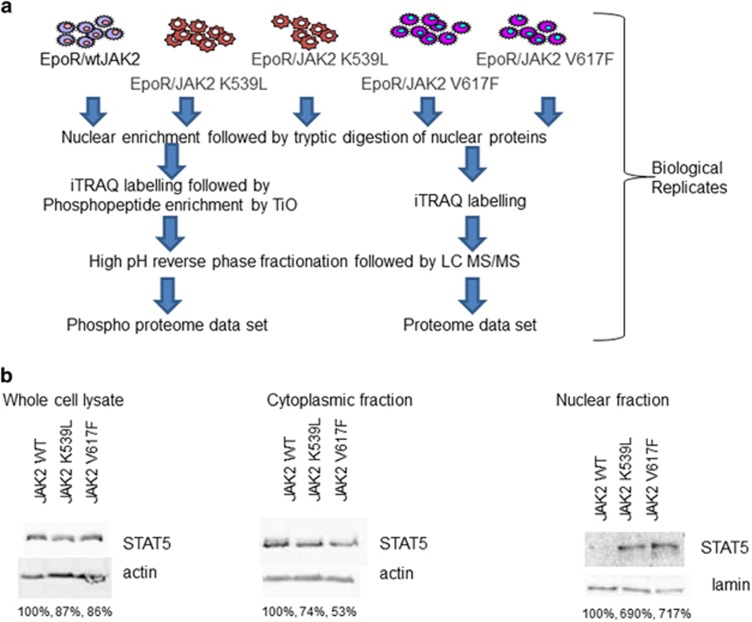
(**a**) A schematic representation of the experimental workflow. Inclusion of duplicate cell lines allows the statistical derivation of a protein change. The experiment (including generation of cell pellets) was performed three times. (**b**) Western blot of STAT5a expression in Ba/F3 whole cell lysates, nuclear and cytoplasmic fractions (actin and lamin shown as loading controls). Protein expression as a percentage of JAK2WT levels are indicated.

**Figure 4:**
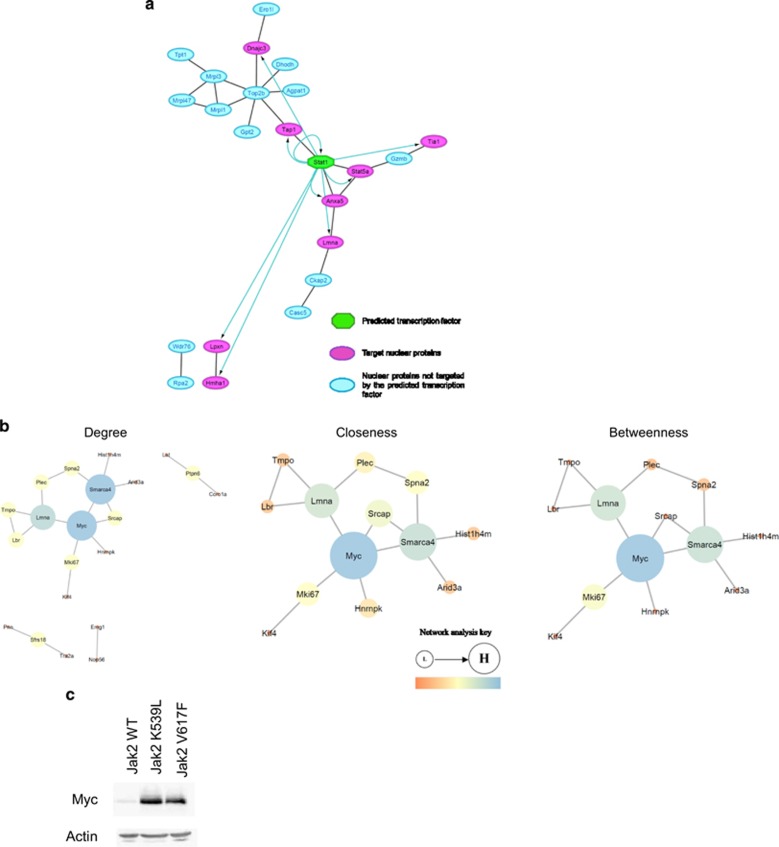
The interactions between the nuclear proteins and proteins corresponding to the phosphopeptides defined as changing were identified using ‘STRING’^45^ and exported to ‘Cytoscape’ software.^46^ Predicted transcription factors and corresponding targets for the proteins (**a**) were identified using the ‘iRegulon’ plugin.^47^ The phosphopeptide interactions were analysed (**b**) using the ‘NetworkAnalyzer’ plugin.^48^ Nodes are sized and coloured according to their centrality values. (**c**) Western blot of MYC expression in Ba/F3 whole cell lysates (actin shown as a loading control).

The changes seen in TGFβ and phospho status of p53, a growth factor and transcription factor, led us to investigate JAK2V617F-induced changes in the proteome and phosphoproteome of the nucleus. Cellular fractionation was undertaken to allow improved quantification of nuclear proteins^15, 27^ and iTRAQ chosen as by their very nature phosphoproteomics data sets rely upon single peptide observations. The multiplex nature of iTRAQ allows the use of duplicate cell lines that via direct comparison allows us to statistically define a change more rigorously (Supplementary Table 1) and include two disease-associated JAK2 mutations (Figure 3a). Peptides derived from nuclear proteins, enriched via subcellular fractionation, were iTRAQ labelled and subject to liquid chromatography/tandem mass spectrometry before and after phosphopeptide enrichment via Ti02 chromatography. A total of 3391 proteins (false discovery rate of 0.14%) were identified and quantified (Supplementary Table 5). Using the criteria described in the methods to determine a change (shown in Supplementary Table 1), 60 proteins were shown to change as a consequence of both JAK2V617F and JAK2K539L expression (Supplementary Table 6). Mutant JAK2 proteins are known to activate the signal transducer and activator of transcription (STAT) pathway but showed no differences in expression in whole cell lysates (Supplementary Table 4); however, STAT5a and STAT1 demonstrate a marked increase in nuclear localisation (Supplementary Table 6). This observation was validated by western blot (Figure 3b) where total expression level of STAT5a can be seen to be equivalent but expression of both JAK2 mutants led to a translocation from the cytoplasm to the nucleus. Indeed, an *in silico* analysis of transcription factors potentially involved in the observed changes in nuclear protein expression identified STAT1 as the major transcription factor responsible (Figure 4a).

Enrichment of nuclear phosphopeptides via Ti0_2_ chromatography allowed the identification and quantification of 2090 phosphopeptides (Supplementary Table 7) and a peptide change defined as for proteins (Supplementary Table 1). Of the 2090 phosphopeptides identified, 38 were shown to change as a consequence of both JAK2V617F and JAK2K539L expression (Supplementary Table 8). Degree network analysis (Figure 4b), designed to identify proteins with high level of interactions within the network of proteins corresponding to the changing phosphopeptides, identified MYC as an important node with a high degree of connectivity, demonstrating its potential as a possible network hub.^28^ MYC was also identified as the node with the highest closeness and betweenness centrality measures (these are measures of the level and importance of its connections) within the major component of the network, demonstrating its high extent of independence^29^ and ability to control the flow within the network,^29, 30^ respectively, thus adding further evidence to its potential important role in the observed changes. In line with these predictions are the fact that phosphorylation of MYC on serine 62 (Supplementary Table 8) has been linked to both MYC activity^31^ and stabilisation.^32^ Western blot analysis of MYC levels in the JAK2V617F- and JAK2K539L-expressing cells show a marked upregulation of MYC protein expression (Figure 4c).

### Pathway disruption in primary material

**Figure 5:**
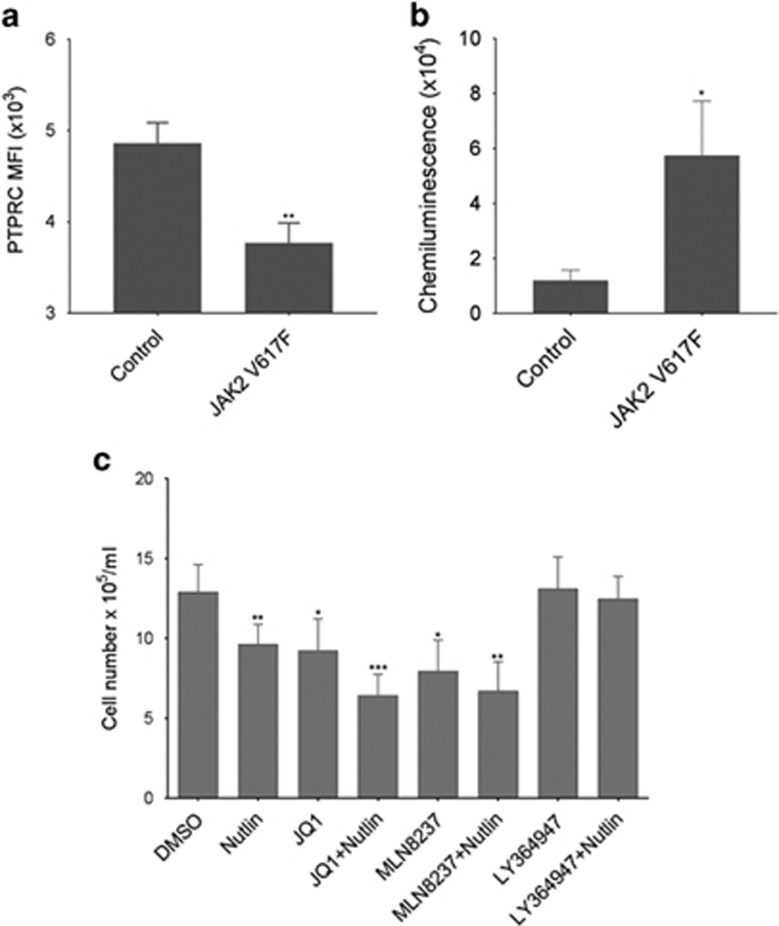
(**a**) Cell surface expression of PTPRC on CD34+ cells isolated from control and JAK2V617F patients was assessed using flow cytometry. Results shown are mean fluorescence intensity±s.e.m. (*n*=6 for controls, *n*=8 for JAK2V617F patients). (**b**) MYC expression was measured using Peggy-Sue (Protein Simple). Expression levels were normalised using actin and the quantification illustrated using the chemiluminescent peak area (*n*=6±s.e.m.). (**c**) The effect of inhibition of MYC (250 nM JQ1), aurora kinase (50 nM MLN8237) and TGFβ receptor (10 μM LY364947) in combination with the activation of p53 (500 nM nutlin) on cell proliferation was assessed over 8 days in liquid culture (mean±s.e.m., *n*=8). The *t*-test results are represented by *P<0.05, **P<0.01, ***P<0.001.

We next sought to confirm our observations in patient material. CD34+ cells were isolated from patients with the JAK2V617F mutation and nondiseased people. In line with our data, PTPRC expression showed a clear reduction in patients expressing JAK2V617F (Figure 5a) whereas MYC displayed an increase in expression (Figure 5b). Having validated some of our proteomic observations, we chose to further investigate the changes in TGFβ secretion, MYC expression and p53 phosphorylation. These three proteins are potential therapeutic targets of particular interest as they are all direct or indirect drug targets with associated clinical trials either in leukaemias or other cancers (for example, Clinicaltrials.gov identifiers NCT01291784, NCT01949883, NCT01164033). Furthermore, we have recently identified a p53-MYC dual hub responsible for many of the BCR/ABL-induced changes in CML and illustrated the fact that targeting these two proteins eliminates the leukaemic stem cell.^33^

### Dual drug treatments increase elimination of the MPN clone

Given the upregulation of MYC and TGFβ and the phosphorylation of p53 on an Aurora kinase consensus sequence, we investigated the possibility of leukaemic cell depletion by a combination of activating p53 in conjunction with inhibition of the other pathways. We treated normal CD34+ haematopoietic cells and their JAK2V617F-positive patient counterparts with a p53 activator (nutlin) in combination with MYC (JQ1) and TGFβ signalling (LY364947) inhibitors. Nutlin inhibits the interaction between Hdm2 and p53, leading to the stabilisation of p53.^34^ JQ1 is a BET bromodomain inhibitor that reduces transcription by disruption of chromatin-dependent signalling^35^ with MYC being shown as a primary target.^36^ Given the increase in p53s212 phosphorylation and our previous results on the effects of aurora kinase on p53s212 phosphorylation,^26^ and hence control of p53-induced differentiation, we also used an aurora kinase (MLN8237) inhibitor.

**Figure 6:**
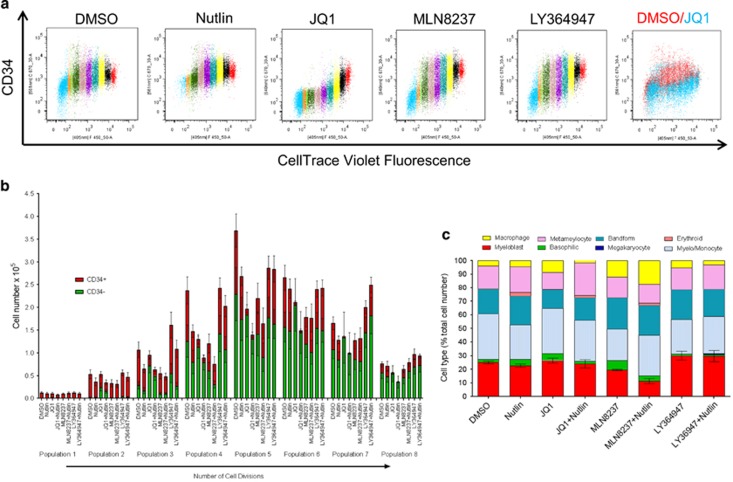
CD34+ cells isolated from JAK2V617F patients were stained using CellTrace. The effect of inhibition of MYC (250 nM JQ1), aurora kinase (50 nM MLN8237) and TGFβ receptor (10 μM LY364947) in combination with the activation of p53 (500 nM nutlin) on cell proliferation and differentiation was assessed over 8 days in liquid culture. (**a**) Representative flow cytometric analysis of CD34 expression in relation to cell division. Each colour represents a cell division. (**b**) Amalgamated results of the effects on cell division and CD34 expression (mean±s.e.m., *n*=8). (**c**) Cells were stained with May-Grunwald-Giemsa and morphology assessed. Data are expressed as the mean (*n*=3) for each cell type categorised as a percentage of the total cells. For presentation purposes error bars (s.e.m.) are shown for the number of cells identified as myeloblasts only.

**Figure 7:**
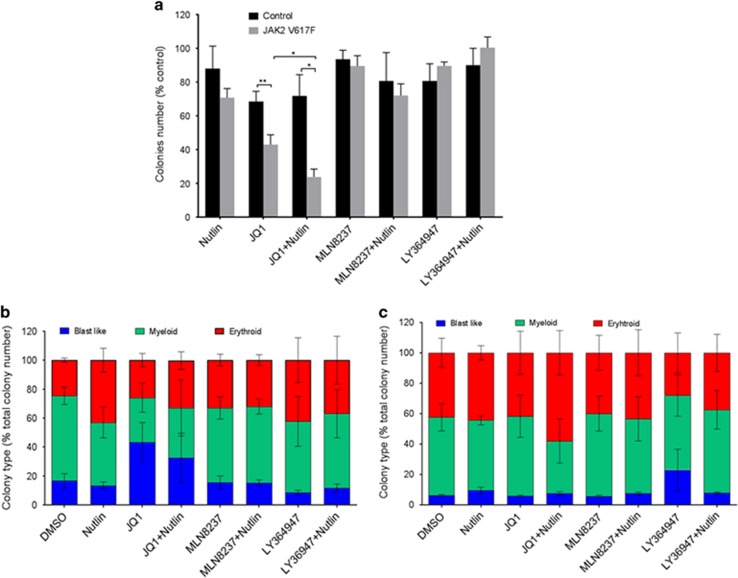
The effect of inhibition of MYC (250 nM JQ1), aurora kinase (50 nM MLN8237) and TGFβ receptor (10 μM LY364947) in combination with the activation of p53 (500 nM nutlin) on the ability of CD34+ cells from JAK2V617F-positive patients and nonleukaemic patients to form colonies in methylcellulose was assessed. Colonies were counted after 7 days (**a**) and the data displayed as the total number of colonies expressed as a percentage of the vehicle control (mean±s.e.m., *n*>3<8). Colony morphology was assessed after 14 days and is displayed as a percentage of colony type (mean±s.e.m., *n*=3) for JAK2V617F patient (**b**) and control patients (**c**). The *t*-test results are represented by *P<0.05, **P<0.01.

**Figure 8:**
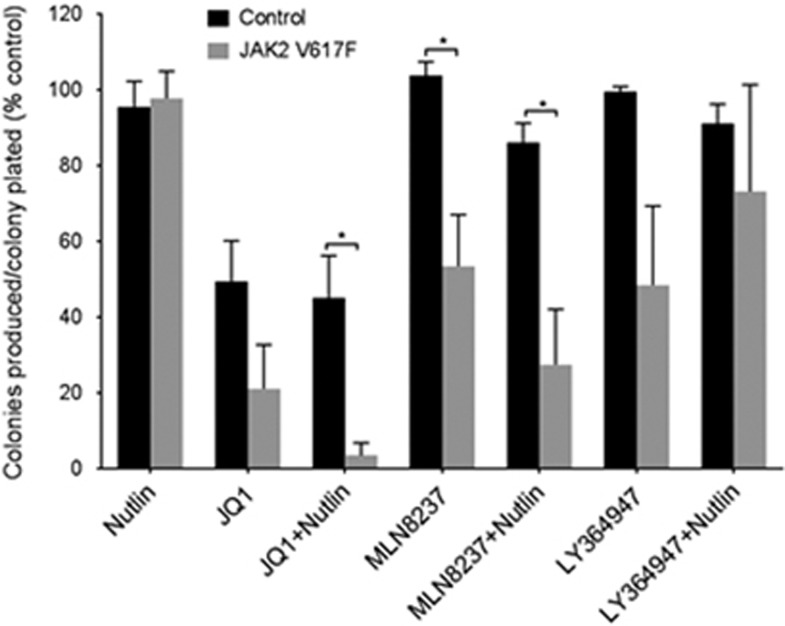
Colonies produced after 7 days were resuspended and replated in methylcellulose. The number of colonies was assessed following 14 days and the data expressed as a percentage of control in relation to the number of colonies produced as a fraction of the colonies plated (mean±s.e.m., *n*=3). The *t*-test results are represented by *P<0.05.

Initial experiments were undertaken in liquid culture. CD34+ cells were isolated from peripheral blood of JAK2V617F-positive patients and labelled with CellTrace to allow analysis of both division and differentiation. Although all treatments except those that contained the TGFβ inhibitor LY364947 reduced cell numbers (Figure 5c), the most marked effect was the rapid loss of CD34+ expression within the first two cell divisions in the presence of JQ1 (Figures 6a and b). We therefore looked at the morphological features of the cells by staining with May-Grunwald-Giemsa after 7 days in culture. The blast cell populations had dissipated in all cultures but the morphological assessment indicated that the development of the cells was only significantly affected by treatments that included the aurora kinase inhibitor, MLN8237 (Figure 6c). The number of blast cells was significantly reduced in the MLN8237 and MLN8237 plus nutlin samples only (*P*=0.01 and 0.007, respectively). Thus, growth and development of CD34+ JAK2V617F-expressing cells are affected by nutlin, JQ1 and MLN8237 but in different ways. JQ1 induced an early loss of a primitive cell marker and reduced cell numbers, whereas MLN8237 reduced cell number while leading to discordant development. Nutlin in combination with either drug enhanced these effects. As depletion of primitive leukaemic cells is the aim of successful treatment strategies, these data led us to examine the possibility that we could use these drugs singly or in combination to critically deplete self-renewing JAK2V617F cells. This was undertaken using haematopoietic colony-forming assays comparing CD34+ cells from JAK2V617F-positive and control patients. Colony numbers from CD34+ enriched cells were assessed at 7 days (Figure 7a). As could be predicted from the rapid loss of CD34+ expression in liquid culture, both JQ1 alone and in combination with nutlin had a significant effect on reducing the number of colonies produced from JAK2V617F CD34+ cells when compared with untreated controls. Critically, these two drugs also showed significant preferential killing of JAK2V617F-positive cells when compared with control patients (Figure 7a). Furthermore, JQ1 and nutlin in combination had a significantly greater effect than either treatment alone. This mirrors our recently reported results in CML where by simultaneously modulating p53-driven apoptosis and MYC-driven differentiation we were able to selectively extinguish the leukaemic stem cell.^33^ No significant effect on colony size was obtained in any condition. Morphological assessment of the cells indicated an increase in the proportion of blast-like cells within the reduced number of colonies produced in the presence of JQ1 (Figures 7b and c). This inferred increased self-renewal of a limited set of colonies. To determine whether this was the case or whether in fact the primitive cells were being extinguished by any of the drug combinations, we next considered the replating potential of cells from each condition. In this experiment JQ1 plus nutlin markedly decreased the replating potential (Figure 8) and as such suggested the drugs affected primitive MPN cells. Our initial results indicating a rapid loss of CD34 expression (Figure 6b) but increase in blast-like cells in the presence of JQ1 (Figure 7b) argued for a JQ1-induced discordant development, but the loss of replating ability (Figure 8) inferred self-renewing cells were being lost from culture. In addition, nutlin markedly ameliorated this effect in a way that almost extinguishes replating cells. Interestingly, despite having no measurable effect on colony number or morphology in the first instance (Figures 7a–c), aurora kinase inhibition (MLN8237) had a marked effect on the replating ability of the MPN cells (Figure 8) that also inferred an effect on primitive cells.

## Discussion

Treatment of PV aims to both alleviate the symptom burden and also prevent transformation to myelofibrosis or secondary acute myeloid leukaemia. Unfortunately, standard therapies are ineffective in preventing the transformation process. The introduction of specific kinase inhibitors to JAK2 promised to be a major advance in the treatment of MPNs. Although the JAK2 inhibitors had significant effects on symptom burden, such as splenomegaly, very few patients showed reduced allele burden.^12, 13^ More recent studies however have reported partial molecular response in up to 10% of patients with longer-term follow-up.^37, 38^ It is envisaged that using drug combinations will improve therapeutic outcome and several clinical trials have been proposed utilising such strategies, for example, JAK2 inhibitors in combination with PIM inhibitors,^39^ deacetylase inhibitors^40^ or combinations of interferon-α and MDM2 antagonists.^41^ To identify such novel partners we undertook a proteomic and phosphoproteomic screen of JAK2V617F-expressing cells. This systematic approach indicated a role for TGFβ, p53 and MYC in the JAK2V617F perturbation of the proteome.

Our observation on p53 disruption was based around increased phosphorylation rather than changes in expression or mutation as classically seen in many cancers. This reflects the situation in the clinical presentation of MPN where p53 mutations are rarely seen at presentation but disruption in the p53 network or actual mutations in p53 are found in >50% MPN secondary leukaemias.^42^ It has been reported that JAK2V617F inhibits the stabilisation of p53 after induction of DNA damage via increases in MDM2 translation such that p53 has been suggested to play a role in disease progression^25^ and offer a valid target in MPN. In fact, these data were used by Lu *et al.*^41^ to devise a potential treatment strategy for MPN by both increasing p53 transcription using interferon-α 2a and inhibiting p53 degradation with an MDM2 antagonist. They were able to demonstrate a significant reduction in allele burden in xenograft models and a clinical trial is now underway (NCT02407080). Our observation on the change in p53 phosphorylation and our earlier work on the aurora kinase control of p53 phosphorylation^27^ led us to investigate the utility of aurora kinase inhibitors in adversely affecting the MPN stem cells. The inhibition of aurora kinase in colony-forming assays showed no effect on cell numbers but did affect the self-renewal/differentiation balance as there was a significant reduction in colony-forming ability of the cells produced in the presence of the inhibitor. This fits with our earlier data suggesting the aurora kinase control of p53 phosphorylation controls embryonic stem cell differentiation.^27^ The lack of effect on colony-forming ability of p53 upregulation alone (treatment with nutlin) is potentially because of the aurora kinase phosphorylation of p53 preventing its activation that would suggest that any therapies aimed at activating p53 may benefit from the simultaneous inhibition of aurora kinase.

Our observation on the JAK2V617F-induced upregulation of MYC fits with other work where MYC-driven increases in ornithine decarboxylase are suggested to be involved in JAK2V617F-induced transformation^43^ and that a combination of PIM inhibitor-driven MYC downregulation and JAK2 inhibition is more effective than either agent alone in suppressing MPN cell growth.^39^ In line with our recent demonstration of elimination of the quiescent stem cell in CML xenograft models,^33^ using a combination of p53 activation and MYC inhibition, our results here indicate the wider potential use of this approach and warrant preclinical studies in murine mutant JAK2 models. Although there appears to be significant therapeutic benefit, it must be remembered that JQ1 is a Brd protein inhibitor^35^ and that in addition to its effects on MYC has been reported to inhibit the transcriptional activity of STAT5^44^ such that the exact molecular mechanism by which we are achieving preferential extinction of MPN cells is still to be determined.

In conclusion, our proteomic study identified perturbation in p53 and MYC pathways and we were able to demonstrate their potential as therapeutic targets in MPN. Our current work is aimed at expanding these observations into preclinical murine models of MPN with a view to rapidly moving to clinical trials in conjunction with our work on CML.^33^
