# Erratum: Proteomic analysis of JAK2V617F-induced changes identifies potential new combinatorial therapeutic approaches

**DOI:** 10.1038/leu.2017.333

**Published:** 2017-12-19

**Authors:** S Pearson, A J K Williamson, R Blance, T C P Somervaille, S Taylor, N Azadbakht, A D Whetton, A Pierce

**Keywords:** Proteomic analysis, Oncogenes, Cancer therapy, Myeloproliferative disease

**Correction to:** *Leukemia* (2017) **31**, 2717–2725; doi:10.1038/leu.2017.143; published online 23 May 2017

This article was originally published under a CC BY-NC-ND 4.0 license, but has now been made available under a CC BY 4.0 license. The PDF and HTML versions of the paper have been modified accordingly.

