# Supplementary material for: Proteomic analysis of JAK2V617F-induced changes identifies potential new combinatorial therapeutic approaches
Source: Leukemia. 2017 Jun 16;31(12):2717–25. doi: 10.1038/leu.2017.143 (PMC5729335; doi:10.1038/leu.2017.143)
Supplement: Supplementary Information [file leu2017143x1.docx]

**Supplementary Methods**

**Cell lines and SILAC labelling**

All cell lines were tested for mycoplasma on a monthly basis. Ba/F3 cells were transfected with MSCV retroviral vector containing Epo receptor (EpoR) or EpoR with either wild type JAK2 or JAK2 mutants K539L or V617F as previously described^(^[^1^](#_ENREF_1)^)^. EpoR expression is required to enable JAK2 mutant transformation in hematopoietic cells^(^[^2^](#_ENREF_2)^)^ and as Ba/F3 cells do not express EpoR there is a requirement to co-express the receptor to produce a model of leukemic transformation. The resultant cell lines were maintained in culture in RPMI with 10% (v/v) horse serum. Ba/F3 and EpoR cells were grown in RPMI with 10% (v/v) horse serum supplemented with 5% mIL-3 (conditioned media from X63-Ag-653 cells). For SILAC Labelling JAK2 WT and JAK2 V617F cells were grown as described above except the horse serum used had been dialysed with a 10,000 Da molecular weight cut off. JAK2 WT cells were cultured in “light” SILAC media containing ^12^C_6_ -Lys and JAK2 V617F expressing cells in “heavy” SILAC media containing ^13^C_6_ ^15^N_2_ –Lys. The heavy lysine contains 13C6 giving a mass difference of approximately 6Da. The cells were cultured in the described media for 5 passages before using mass spectrometry to confirm complete protein labelling with heavy lysine before proceeding with quantification experiments. Cells had similar growth characteristics (Supplementary Figure 1).

**Identification of SILAC labelled proteins.**

SILAC labelled peptides were analysed on a LTQ Orbitrap Velos (Thermo Fisher). Peptide was loaded onto Acclaim PepMap µ-Precolumns (500µm×5mm) and flow was set to 30µl/min of 3%[v/v] acetonitrile, 0.1%[v/v] formic acid for 5min. Analytical separation of the peptides was performed using Acclaim PepMap RSLC C18 Columns (2 µm, 75 µm×500 mm). Peptides were separated over a 91 min solvent gradient from 3%[v/v] acetonitrile, 0.1%[v/v] formic acid to 40%[v/v] acetonitrile, 0.1%[v/v] formic acid. Data were acquired using an IDA method. For each cycle, one full MS scan of m/z 300–1700 was acquired at a resolution of 60,000 at m/z 400 with an automatic gain control target of 1x10^6^. Each full scan was followed by the selection of the 20 most intense ions, CID and MS/MS analysis was performed in the LTQ at a normal resolution. Selected ions were excluded from further analysis for 60s. Ions with an unassigned charge or a charge of +1 were rejected.

**Preparation of cellular nuclei**

Nuclear proteins were enriched using a kit from Active Motif (Rixensart, Belgium) with some modifications. Briefly, 1 x 10^7^ cells in mid log phase growth were washed in ice cold Hanks buffer with protein phosphatase inhibitors and incubated in hypotonic buffer (750µl) as per manufacturer’s instructions. The preparation was then incubated on ice for 20 minutes in 50µl TEAB (0.5M), 0.05% (w/v) SDS and 0.5µl of protease inhibitor cocktail (Active Motif) and 2µl/ml Benzonase (Novagen, UK). The preparations were mixed, centrifuged for 10minutes at 14,000g and the supernatant (nuclear fraction) recovered. The quality of the preparations was confirmed using western blot analysis for nuclear (lamin A/C) and cytosolic (α tubulin) standard marker proteins.

# Isobaric tagging for 8 channel analysis and liquid chromatography

# 100 µg of nuclear proteins from each Ba/F3 cell line were reduced, alkylated, and subjected to tryptic hydrolysis prior to labelling with 8 channel isobaric tagging iTRAQ reagent (AB Sciex, UK) in 1M TEAB, according to the manufacturer’s instructions (in all experiments isobaric labelling exceeded 98% of total identified peptides using mass spectrometry). Two biological replicates of each cell mutant JAK2 cell line were labelled to provide an internal control for the experiment and assess biological variation. The experiment (including generation of cell pellets) was performed four times for the proteome and twice for the phosphopeptide assessment. Isobarically tagged peptides were then taken forward for analysis or subjected to phosphopeptide enrichment on TiO_2_ columns. 100 µg of nuclear iTRAQ labelled peptide mixtures were re-suspended in 150µl of lactate loading buffer (240mg/mL lactate in 80% (v/v) acetonitrile, 1% (v/v) Trifluoro-acetic acid (TFA). TiO_2_ columns (TopTip, Glygen Corp, USA) were equilibrated with 150µl of lactate loading buffer then the samples loaded. The tip was washed twice with 60µl of lactate loading buffer and four times with 60µl of wash buffer (80% (v/v) acetonitrile, 5% (v/v) TFA). Bound peptides were then recovered by elution in 60µl of elution buffer (ammonium water [20µl NH_3_ in 980ul H_2_O], pH 10.5). Samples were then concentrated to a few µl in a SpeedVac prior to RP-LC-MS/MS.

**High pH Reversed Phase chromatography**

Prior to reversed phase LC-MS/MS peptides were fractionated off line using a reversed phase chromatography column (Fortis technologies, C18 3μm 100/4.6 Reversed phase column) at a high pH using an LC Packings Ultimate LC system. The gradient was run at 700 µl/min using initially 99.5% high pH buffer A (0.1% Ammonium hydroxide, adjusted to pH 10.5 with formic acid) 0.5% high pH buffer B (0.1% Ammonium hydroxide, 99.9% acetonitrile). Over a 30 minute time period high pH buffer B was increased to 50%, followed by a 4 minute time period to increase high pH buffer B to 75%. The concentration of high pH buffer B (75%) was held for 4 minutes then reduced back down to 0.5%. Fifteen second fractions were collected for the duration of the gradient, the volume of each fraction was then decreased under vacuum.

**Identification of iTRAQ labelled peptides.**

Peptides were identified by RP-LC-MS/MS on a 5600 triple ToF mass spectrometer (AB Sciex, UK). Dried peptide fractions were re-suspended in 15 µl of 3% (v/v) acetonitrile, 0.1% (v/v) formic acid and 20 mM citric acid. For each analysis, 5 µl of the peptide sample was loaded onto a nanoACQUITY UPLC Symmetry C18 Trap, 5 µm, 180 µm×20 mm and flow was set to 15 µl/min of 3% (v/v) acetonitrile, 0.1% (v/v) formic acid and 20 mM citric acid for 5 min. Analytical separation of the peptides was performed using nanoACQUITY UPLC BEH C18 Column, 1.7 µm, 75 µm×250 mm. Briefly, peptides were separated over a 91 min solvent gradient from 3% (v/v) acetonitrile,0.1% (v/v) formic acid to 40% (v/v) acetonitrile,0.1% (v/v) formic acid, on-line to a 5600 Triple ToF mass spectrometer (AB Sciex, UK). Data was acquired using an information dependent acquisition (IDA) protocol where, for each cycle, the 20 most abundant multiply charged peptides (2^+^ to 4^+^) above a 150 count threshold in the MS scan with m/z between 400 and 2000. Each peptide was dynamically excluded (±50 mmu) for 90 seconds.

**Data analysis**

MS data from SILAC labelled samples was processed and quantified using MaxQuant (version 1.2.2.5) software using the ENSEMBL mouse protein database (release 63). Data were analysed with the following parameters: Carbamidomethylation (C) as a fixed modification, oxidation (M) and acetylation (N-term) as variable modifications, trypsin digestion with two missed cleavage sites, mass tolerance of 0.5 kDa, minimum peptide length 6 amino acids and protein ratios based on a minimum of two peptide ratios. Protein and peptide FDR were set at 0.01.

MS data from iTRAQ labelled samples was processed by a ‘Thorough’ search against the ENSEMBL mouse database (release 63) using ProteinPilot 3 software (Paragon version 3.0.0.0, 113442) with default settings including the allowance of one missed or nonspecific cleavage (AB SCIEX, USA), MMTS and 8 plex iTRAQ fixed modifications. For a list of all variable modifications see ProteinPilot 3. Phosphopeptides identified with a confidence of >20 were included in the analysis. ProteinPilot quantification was used for the proteome experiments. For the phosphopeptide experiments, inferred phosphopeptide entities were selected from the ProteinPilot search results. The calculation used by ProteinPilot to derive a weighted average ratio for proteins was used to derive an average ratio where multiple spectra were found for a given distinct phosphoentity (that is, a distinct sequence and set of modified amino acids). The false positive rate for phospho peptides was 0.24%. A protein/phosphopeptide change was defined as any protein having an iTRAQ reporter ion-based relative quantification ratio outside the range in which 95% of protein/phosphopeptide ratios for the internal replicate are found and a p-value of 0.05 or less. This "significance interval" was determined for each experimental run and accounts for the technical and biological variation seen in each run. The values defining a change for each experiment are shown in Supplementary Table 1.

**Network of protein-protein interactions**

The interactions between the proteins defined as changing were identified using the STRING software ^(^[^3^](#_ENREF_3)^)^. The identification of these functional links between proteins was based on neighbourhood in the genome, gene fusion, co-occurrence across genomes, co-expression, experimental data, databases, textmining and homology. Interactions were extracted and imported to “Cytoscape” software ^(^[^4^](#_ENREF_4)^)^ for further analysis and visualisation.

**Prediction of transcription factors and targets**

To predict the presence of potential transcription factors (TFs) involved in the observed protein changes in the nucleus, corresponding network of protein-protein interactions was analysed using the “iRegulon” plugin ^(^[^5^](#_ENREF_5)^)^ in “Cytoscape” software ^(^[^4^](#_ENREF_4)^)^. The prediction for the submitted list of differentially expressed genes was computed following motif and track discovery, motif2TF mapping and target detection ^(^[^5^](#_ENREF_5)^)^. iRegulon analysis is based on the use of a compiled database of position weight matrices (PWMs) libraries. These libraries are constructed through examination of the presence of candidate motifs by inspecting and scoring the cis-regulatory sequences around the Transcription Start Site (TSS) of each gene in the human genome. These findings are then ranked and combined by equivalent analysis carried out in other species’ genomes. The rankings are then integrated to formulate libraries of PWMs, which are then used to identify motifs that are highly enriched in relation to the submitted gene list. Enrichment is represented by the Normalised Enrichment Score (NES) and is calculated using the Area Under the Curve (AUC) for Receiver Operating Characteristic (ROC) curve (cumulative recovery curve) ^(^[^5^](#_ENREF_5)^)^. The potential transcription factors that are associated with the enriched motifs are then detected by motif2TF mapping. Following this the corresponding target genes of the detected transcription factors are identified ^(^[^5^](#_ENREF_5)^)^. Results include enriched motif rankings and predicted transcription factors. Motifs are ranked using NES and are presented along with the AUC for ROC curve that is used to calculate enrichment score, Cluster code of the enriched motifs that are clustered by similarity, number of unique target genes for the given motif and number of transcription factors associated by the motif2TF algorithm to the given motif. To perform iRegulon analysis for this study, the gene symbols corresponding to the 23 interacting differentially expressed nuclear proteins were submitted for analysis. Parameters were set as default for *Ranking*, *Recovery* and *TF prediction* steps. Following this analysis an updated protein-protein interaction network was constructed using the predicted interactions between the most probable transcription factor and the identified targets genes.

**Network statistical analysis**

To assess centrality within the network ^(^[^6^](#_ENREF_6)^)^, statistical analysis was conducted for the network of proteins corresponding to the phosphopeptides defined as changing. Analysis was performed using the “NetworkAnalyzer” plugin ^(^[^7^](#_ENREF_7)^)^ in “Cytoscape” software. Node “degree”, “closeness centrality” and “betweenness centrality” statistics were computed for the network of interacting proteins. Degree of a node represents the extent of its connectivity within the network ^(^[^8^](#_ENREF_8)^)^ by indicating the number of interactions it has with other nodes, and can be used to assess its potential to be recognised as a possible network hub ^(^[^9^](#_ENREF_9)^)^. “Closeness centrality” refers to the inverse of the average of the shortest path distances between a given node and all other nodes in the network ^(^[^6^](#_ENREF_6)^,^ [^10^](#_ENREF_10)^)^. Closeness centrality demonstrates the extent of independence of a node within the network. Nodes with high closeness centrality are situated close to many other nodes within the network, and communication with other nodes via these nodes does not necessitate reliance upon the use of many intermediary nodes. Distance refers to the number of edges in a path that connects two nodes ^(^[^6^](#_ENREF_6)^,^ [^8^](#_ENREF_8)^)^. Shortest path between two nodes refers to a path that involves passing through the smallest number of edges to travel between those two nodes ^(^[^6^](#_ENREF_6)^,^ [^8^](#_ENREF_8)^)^. “Betweenness centrality” of a selected node refers to the ratio of the number of shortest paths between a pair of nodes in the network that pass through the selected node, to the total number of shortest paths that connect the pair of nodes in the network ^(^[^11^](#_ENREF_11)^)^. Betweenness centrality indicates the ability of a node to control the flow (or interactions) within the network ^(^[^6^](#_ENREF_6)^,^ [^11^](#_ENREF_11)^,^ [^12^](#_ENREF_12)^)^.

To conduct network analysis for this protein-protein interaction network of the study, disconnected nodes were not included in the analysis. Network was assumed to contain undirected edges. Node degree, closeness centrality and betweenness centrality statistics were computed for the interacting proteins. To enable the identification of proteins with possible significant role in the protein-protein interaction network of the observed changes, results were compared across all the analysed nodes to detect those with high degree, closeness centrality and betweenness centrality parameter values. Following these analyses, computed statistical measurements were interpolated and illustrated on the network. Nodes with low parameter values were represented by smaller size circles and those with high parameter values were represented by larger size circles. Nodes were also coloured according to their corresponding parameter values.

**Protein expression using Peggy-Sue**

Cells were washed three times in PBS and incubated in lysis buffer (20 mM HEPES pH 7.9, 2 mM MgCl2, 137 mM NaCl, 10% Glycerol, 1% NP-40, 2 mM EDTA, 1 mM Na3VO4, 10 mM NaF, X1 protease inhibitor (Sigma, P8340), X1 Phosphatase Inhibitor Cocktail 2 (Sigma, P5726), Phosphatase Inhibitor Cocktail 3 (Sigma, P0044), 0.5 U/µL Benzonase (Millipore, 70664)) at 2 x 10^6^ per 50 µL for one hour on ice with agitation at 10 minute intervals. Total protein was calculated by Bradford assay using Bio-Rad Protein Assay (Bio-Rad, 500-0006) with levels read at 620/405 nm. Cellular p53 and actin were measured on a Peggy-Sue system (Protein Simple, 004-800) using a 12-230 kDa Master Kit (Protein Simple, PS-MK06) as per manufacturer’s instructions. Protein was loaded at 400 ng/µL per well and separated using default machine settings. Antibodies used were p53 (Cell Signalling, 9282) at 1:100 and actin (Cell Signalling, 4968) at 1:100 in antibody diluent (Protein Simple, 042-514). Both antibodies were multiplexed in individual capillaries giving an internal loading control. Quantification of p53 was carried out using chemiluminescent peak area with normalisation to actin.

**Primary cell colony forming assays**

Use of human tissue was in compliance with the ethical and legal framework of the Human Tissue Act. Experiments had ethical approval from the NRES committee of the regional NHS health research authority (14/LO/0489). Primary JAK2 V617F positive samples were obtained from the Manchester Cancer Research Centre Biobank (HTA 30004) following authorisation by the Tissue Biobanks scientific sub committee. Control samples were CD34+ mobilized cells surplus to requirements from patients undergoing chemotherapy and autologous transplantation for lymphoma. Written informed consent was obtained for all samples. The CD34+ cell population was enriched using CliniMACS (Miltenyi Biotec) according to standard protocols. Colony forming assays were performed by plating CD34+ cells in methylcellulose complete media (R&D systems) supplemented with 2u/ml EPO at a density of 3000cells/ml. 0.2ml was dispensed into each of 4 wells of a 24 well plate. Plates were incubated at 370C in 5% CO2 /5% O2 for 7 days before the number of colonies were counted. To assess retention of self-renewal capacity the resulting colonies at day 7 were replated in methylcellulose complete media: One well (0.2ml) was resuspended in 1ml PBS, an aliquot removed centrifuged and the resulting cell pellet resuspended in methylcellulose (1ml) complete media supplemented with EPO. Cells were plated and incubated as described above with the resulting colonies counted at 14 days.

**References**

1. Evans CA, Pierce A, Winter SA, Spooncer E, Heyworth CM, Whetton AD. Activation of Granulocyte-Macrophage Colony-Stimulating Factor and Interleukin-3 Receptor Subunits in a Multipotential Hematopoietic Progenitor Cell Line Leads to Differential Effects on Development. *Blood* 1999; **94**(5)**:** 1504-1514.

2. Scott LM, Tong W, Levine RL, Scott MA, Beer PA, Stratton MR*, et al.* JAK2 Exon 12 Mutations in Polycythemia Vera and Idiopathic Erythrocytosis. *The New England journal of medicine* 2007; **356**(5)**:** 459-468.

3. Szklarczyk D, Franceschini A, Wyder S, Forslund K, Heller D, Huerta-Cepas J*, et al.* STRING v10: protein-protein interaction networks, integrated over the tree of life. *Nucleic Acids Research* 2015 Jan; **43**(D1)**:** D447-D452.

4. Shannon P, Markiel A, Ozier O, Baliga NS, Wang JT, Ramage D*, et al.* Cytoscape: A software environment for integrated models of biomolecular interaction networks. *Genome Research* 2003 Nov; **13**(11)**:** 2498-2504.

5. Janky R, Verfaillie A, Imrichova H, Van de Sande B, Standaert L, Christiaens V*, et al.* iRegulon: From a Gene List to a Gene Regulatory Network Using Large Motif and Track Collections. *Plos Computational Biology* 2014 Jul; **10**(7).

6. Freeman LC. CENTRALITY IN SOCIAL NETWORKS CONCEPTUAL CLARIFICATION. *Social Networks* 1979; **1**(3)**:** 215-239.

7. Assenov Y, Ramirez F, Schelhorn SE, Lengauer T, Albrecht M. Computing topological parameters of biological networks. *Bioinformatics* 2008 Jan; **24**(2)**:** 282-284.

8. Barabasi A-L, Oltvai ZN. Network biology: understanding the cell's functional organization. *Nat Rev Genet* 2004; **5**(2)**:** 101-113.

9. Hsing M, Byler KG, Cherkasov A. The use of Gene Ontology terms for predicting highly-connected 'hub' nodes in protein-protein interaction networks. *Bmc Systems Biology* 2008 Sep; **2**.

10. Doncheva NT, Assenov Y, Domingues FS, Albrecht M. Topological analysis and interactive visualization of biological networks and protein structures. *Nat Protocols* 2012; **7**(4)**:** 670-685.

11. Barthélemy M. Betweenness centrality in large complex networks. *The European Physical Journal B* 2004; **38**(2)**:** 163-168.

12. Opsahl T, Agneessens F, Skvoretz J. Node centrality in weighted networks: Generalizing degree and shortest paths. *Social Networks* 2010 Jul; **32**(3)**:** 245-251.

**Supplementary Figures**

**Supplementary Figure 1**

Cell proliferation was assessed using the WST-1 cell proliferation kit (Roche Diagnostic). Cells were set up at 2x10^4^/ml in triplicate and proliferation measured according to manufactures instructions at 24 hrs. Absorbance was measured at 450/620nm following 4hr incubation with the wst reagent. Data are shown as mean+/- SEM n=4.

**Supplementary Figure 2**

Histogram showing the distribution of (log_2_) protein quantification ratios of all proteins identified in the SILAC data set.

**Supplementary Figure 3**

Histogram showing the (log2) distribution of protein quantification ratios for all proteins identified in each iTRAQ experiment.

**Supplementary Figure 4**


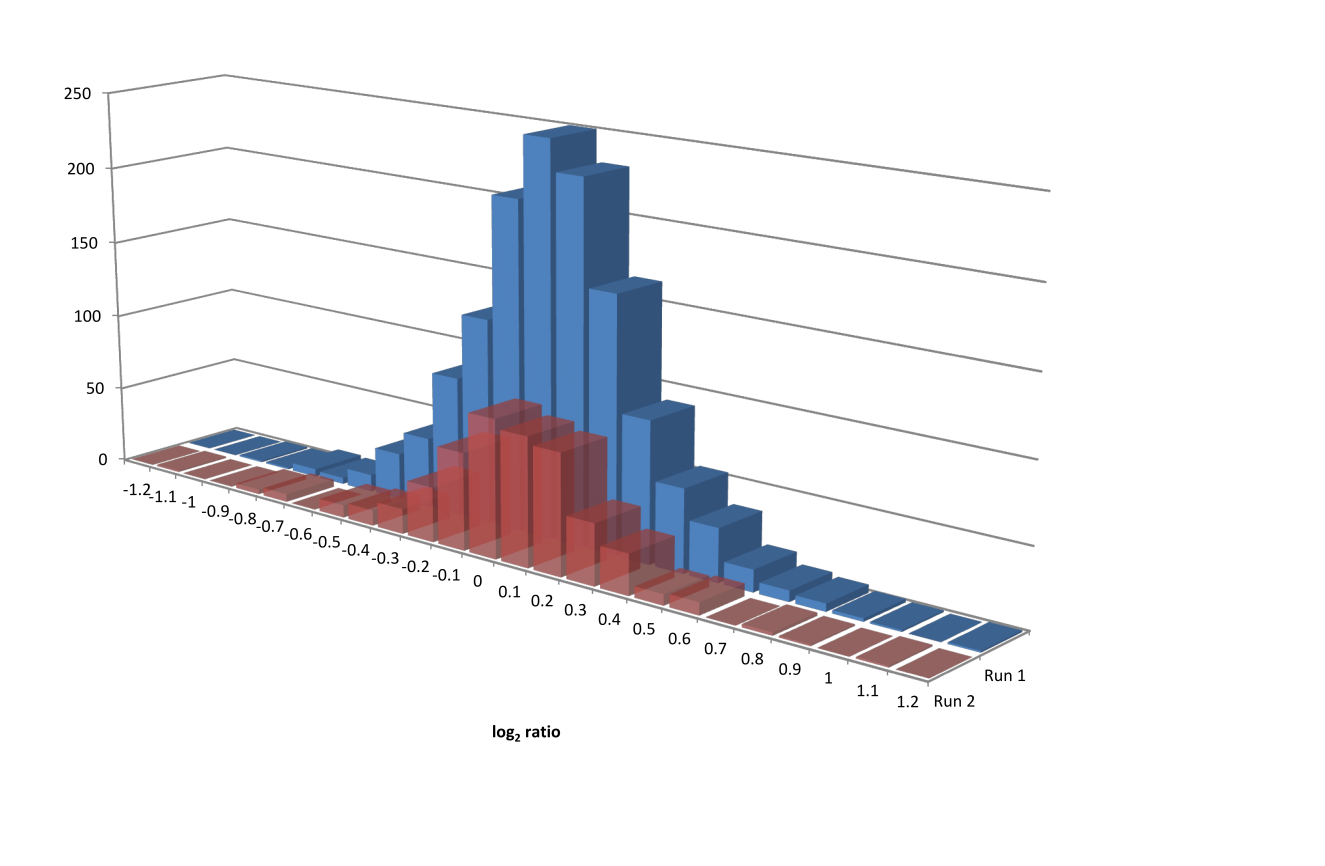


Histogram showing the (log2) distribution of phosphopeptide quantification ratios for all phosphopeptides identified in each iTRAQ experiment.

**Supplementary Tables**

**Supplementary Table 1 – 95% significance interval**

|  | **95% significance interval defining a change** | | | |
| --- | --- | --- | --- | --- |
|  | **Run1** | **Run 2** | **Run 3** | **Technical repeat Run 3** |
| Proteome | >1.40 to < 0.71 | >1.32 to < 0.76 | >1.20 to < 0.83 | >1.22 to < 0.82 |
| Phospho Proteome | >1.54 to < 0.65 | >1.43 to < 0.70 | No run undertaken | No run undertaken |

Table showing the ratios employed to define a change in the nuclear proteome and phosphoproteome data sets. These are the values that 95% of ratios for the internal replicate lie between. This "significance interval" is individually determined for each experimental run and attempts to account for the technical and biological variation seen in each run.

**Supplementary Table 2 – Antibodies used in the study**

| **Antibody** | **Procedure** | **Company** | **Dilution factor** |
| --- | --- | --- | --- |
| β-actin | Western blot | Sigma-Aldrich (A5060) | 1/500 |
| lamin A/C | Western blot | Cell Signaling Technology(#2032) | 1/1000 |
| α-tubulin | Western blot | Santa Cruz (sc-5286) | 1/1000 |
| PTPRC | Western blot | BD Pharmingen(610266) | 1/500 |
| MYC | Western blot | Cell Signaling Technology(#2276) | 1/500 |
| STAT5 | Western blot | Cell Signaling Technology(#9352) | 1/500 |
| CD34+ | Flow cytometry | eBioscience (17-0349) | 1/200 |
| p53 | Simple Western | Cell Signaling Technology(#9282) | 1/100 |
| β-actin | Simple Western | Cell Signaling Technology(#4968) | 1/100 |

**Supplementary Table 4. Proteins whose expression is altered by the expression of JAK2V617F.** Proteins shown are those that are defined as changing. To be called as changing a protein must have associated with it 3 or more peptides and have a ratio of 2 or greater.

| **Accession** | **Gene Symbol** | **Peptides used for Quant** | **JAK2V617F : JAK2WT Ratio** |
| --- | --- | --- | --- |
| ENSMUSP00000075690 | Serpinb1a | 18 | 0.09 |
| ENSMUSP00000083971 | Emr1 | 3 | 0.12 |
| ENSMUSP00000000476 | Pdgfra | 5 | 0.15 |
| ENSMUSP00000077099 | Dock10 | 9 | 0.21 |
| ENSMUSP00000094083 | Csf2rb2 | 3 | 0.22 |
| ENSMUSP00000026546 | Adam8 | 3 | 0.26 |
| ENSMUSP00000034874 | Cyp11a1 | 9 | 0.28 |
| ENSMUSP00000052020 | Flnb | 52 | 0.28 |
| ENSMUSP00000084882 | Gda | 3 | 0.29 |
| ENSMUSP00000099853 | Cd47 | 3 | 0.29 |
| ENSMUSP00000105200 | Epb4.1l1 | 3 | 0.32 |
| ENSMUSP00000027393 | Bard1 | 3 | 0.34 |
| ENSMUSP00000110707 | Fam107b | 9 | 0.34 |
| ENSMUSP00000042028 | Apobr | 3 | 0.34 |
| ENSMUSP00000109456 | Pcx | 9 | 0.35 |
| ENSMUSP00000028135 | Fam129b | 3 | 0.35 |
| ENSMUSP00000038877 | 2810417H13Rik | 4 | 0.37 |
| ENSMUSP00000100862 | Cd63 | 3 | 0.37 |
| ENSMUSP00000067685 | Iqgap2 | 25 | 0.37 |
| ENSMUSP00000074729 | Plekhg3 | 4 | 0.38 |
| ENSMUSP00000026613 | Gusb | 3 | 0.38 |
| ENSMUSP00000091409 | Arl14ep | 3 | 0.38 |
| ENSMUSP00000059033 | Hip1 | 4 | 0.39 |
| ENSMUSP00000107667 | Ptprc | 11 | 0.39 |
| ENSMUSP00000086795 | Lgals1 | 5 | 0.40 |
| ENSMUSP00000020448 | Irak3 | 4 | 0.41 |
| ENSMUSP00000108913 | Tfeb | 4 | 0.41 |
| ENSMUSP00000104652 | Lima1 | 11 | 0.42 |
| ENSMUSP00000124793 | Galnt6 | 3 | 0.43 |
| ENSMUSP00000028239 | Gsn | 5 | 0.43 |
| ENSMUSP00000085253 | Rab44 | 4 | 0.44 |
| ENSMUSP00000110644 | Sema4c | 3 | 0.45 |
| ENSMUSP00000020107 | Atp2b1 | 5 | 0.46 |
| ENSMUSP00000102543 | Dnajc6 | 3 | 0.46 |
| ENSMUSP00000102098 | Eef2k | 4 | 0.47 |
| ENSMUSP00000126024 | Cdk6 | 3 | 0.47 |
| ENSMUSP00000100911 | Osbpl8 | 6 | 0.47 |
| ENSMUSP00000130626 | Dag1 | 4 | 0.47 |
| ENSMUSP00000051645 | Atp13a3 | 3 | 0.49 |
| ENSMUSP00000021726 | Adssl1 | 3 | 0.50 |
| ENSMUSP00000055803 | Glb1 | 4 | 0.51 |
| ENSMUSP00000099703 | Trpv2 | 3 | 0.52 |
| ENSMUSP00000002640 | Scin | 12 | 0.52 |
| ENSMUSP00000095082 | Rcsd1 | 4 | 0.53 |
| ENSMUSP00000034929 | Lactb | 3 | 0.54 |
| ENSMUSP00000071643 | Osbpl3 | 4 | 0.54 |
| ENSMUSP00000115822 | Fam129a | 7 | 0.55 |
| ENSMUSP00000097519 | Mical1 | 4 | 0.55 |
| ENSMUSP00000102050 | Arhgap17 | 6 | 0.55 |
| ENSMUSP00000097100 | Hmha1 | 9 | 1.96 |
| ENSMUSP00000027379 | Xrcc5 | 5 | 1.97 |
| ENSMUSP00000002678 | Tgfb1 | 4 | 1.99 |
| ENSMUSP00000098901 | Mat2b | 5 | 2.05 |
| ENSMUSP00000128714 | Eno3 | 6 | 2.05 |
| ENSMUSP00000137520 | Gpr56 | 5 | 2.06 |
| ENSMUSP00000080949 | Mdc1 | 6 | 2.10 |
| ENSMUSP00000066556 | 9830001H06Rik | 3 | 2.13 |
| ENSMUSP00000082018 | Arrb2 | 5 | 2.17 |
| ENSMUSP00000021611 | Pitrm1 | 18 | 2.17 |
| ENSMUSP00000004565 | Ralb | 4 | 2.20 |
| ENSMUSP00000079991 | Plcg2 | 15 | 2.24 |
| ENSMUSP00000126622 | Zyx | 5 | 2.31 |
| ENSMUSP00000033427 | Sash3 | 4 | 2.34 |
| ENSMUSP00000045890 | Ubash3a | 3 | 2.35 |
| ENSMUSP00000029266 | Anxa5 | 7 | 2.43 |
| ENSMUSP00000043865 | Ubash3b | 3 | 2.60 |
| ENSMUSP00000075950 | Tes | 5 | 2.64 |
| ENSMUSP00000076111 | Zeb2 | 4 | 2.74 |
| ENSMUSP00000066715 | Mosc2 | 4 | 2.81 |
| ENSMUSP00000104952 | Lcp2 | 5 | 2.86 |
| ENSMUSP00000048229 | Lyplal1 | 5 | 3.07 |
| ENSMUSP00000120452 | Hacl1 | 3 | 3.36 |
| ENSMUSP00000079306 | Hspa2 | 6 | 4.59 |
| ENSMUSP00000021028 | Itgb3 | 3 | 4.94 |
| ENSMUSP00000091317 | Pls1 | 3 | 5.04 |
| ENSMUSP00000120045 | Mrvi1 | 3 | 5.57 |
| ENSMUSP00000135294 | Fbxw10 | 3 | 6.29 |
| ENSMUSP00000012348 | Gstm2 | 4 | 6.55 |
| ENSMUSP00000099411 | Tgm2 | 7 | 7.95 |
| ENSMUSP00000104825 | Ifi47 | 6 | 8.05 |
| ENSMUSP00000015581 | Gzmb | 5 | 12.22 |
| ENSMUSP00000103393 | Hemgn | 4 | 18.39 |
| ENSMUSP00000023072 | Parvb | 5 | 20.59 |
| ENSMUSP00000028522 | Itga6 | 4 | 24.11 |

**Supplementary Table 6. Nuclear Proteins whose expression is altered by the expression of JAK2V617F and JAK2K539L**

Proteins shown are those that are defined as changing by the parameters outlined. To be called as changing in one run a protein must have a ratio outside the range in which 95% of protein ratios for the internal replicate are found and a p-value of 0.05 or less. The ratio column contains an average of the ratios across multiple experiments. This is supported by the averaged confidence, ‘’significance’’, which indicates how closely the experiments agree. This must be 0.90 or above for the protein to appear in the list. The confidence for each experiment is the proportion of ratios in the internal replicate that the ratio in question is outside of. (So the value for an ‘Up’ call is between 0.95 and 0.99 inclusive but an average value less than 0.95 still indicates close agreement across multiple experiments). This gives a normalised value between -0.99 and 0.99 for each experiment. A value of greater magnitude indicates more agreement between experiments supporting the change call.

|  |  | **JAK2V617F:JAK2WT** | | **JAK2K539L:JAK2WT** | |
| --- | --- | --- | --- | --- | --- |
| **Accession** | **Gene symbol** | **Significance of change** | **Ratio** | **Significance of change** | **Ratio** |
| ENSMUSP00000029266 | Anxa5 | -0.98 | 0.62 | -0.95 | 0.72 |
| ENSMUSP00000025337 | Diap1 | -0.98 | 0.63 | -0.93 | 0.77 |
| ENSMUSP00000017629 | Top2b | -0.95 | 0.77 | -0.93 | 0.79 |
| ENSMUSP00000029699 | Lmna | -0.94 | 0.73 | -0.97 | 0.70 |
| ENSMUSP00000106519 | Tpt1 | -0.94 | 0.65 | -0.93 | 0.74 |
| ENSMUSP00000015581 | Gzmb | 0.97 | 1.71 | 0.96 | 2.59 |
| ENSMUSP00000097100 | Hmha1 | -0.95 | 0.53 | -0.98 | 0.53 |
| ENSMUSP00000097444 | Prep | -0.94 | 0.70 | -0.91 | 0.75 |
| ENSMUSP00000022378 | Ero1l | -0.92 | 0.52 | -0.97 | 0.51 |
| ENSMUSP00000007257 | Clic1 | -0.92 | 0.74 | -0.93 | 0.76 |
| ENSMUSP00000025835 | Cpt1a | -0.99 | 0.64 | -0.94 | 0.74 |
| ENSMUSP00000066743 | Stat1 | 0.98 | 1.72 | 0.94 | 1.57 |
| ENSMUSP00000033289 | Stim1 | -0.97 | 0.68 | -0.98 | 0.68 |
| ENSMUSP00000039518 | Ckap2 | 0.97 | 1.34 | 0.93 | 1.25 |
| ENSMUSP00000121182 | Faf2 | -0.97 | 0.59 | -0.90 | 0.78 |
| ENSMUSP00000099621 | Rpa2 | 0.96 | 1.25 | 0.91 | 1.18 |
| ENSMUSP00000034949 | Csnk1g1 | -0.94 | 0.76 | -0.93 | 0.79 |
| ENSMUSP00000067680 | Klf13 | 0.99 | 1.67 | 0.98 | 1.57 |
| ENSMUSP00000117299 | Haus2 | 0.99 | 1.58 | 0.98 | 1.48 |
| ENSMUSP00000102980 | Stat5a | 0.99 | 2.22 | 0.98 | 1.94 |
| ENSMUSP00000084679 | Abi2 | 0.99 | 1.82 | 0.99 | 1.79 |
| ENSMUSP00000090897 | Hba-a1 | 0.99 | 2.08 | 0.99 | 3.65 |
| ENSMUSP00000031866 | Mtpn | -0.99 | 0.36 | -0.99 | 0.48 |
| ENSMUSP00000046856 | Lss | -0.99 | 0.47 | -0.97 | 0.69 |
| ENSMUSP00000008594 | Nutf2 | -0.99 | 0.49 | -0.97 | 0.71 |
| ENSMUSP00000022734 | Dnajc3 | -0.99 | 0.51 | -0.99 | 0.53 |
| ENSMUSP00000035992 | Gga1 | -0.99 | 0.70 | -0.97 | 0.80 |
| ENSMUSP00000019726 | Plin3 | -0.99 | 0.62 | -0.93 | 0.77 |
| ENSMUSP00000001046 | S100a4 | -0.99 | 0.26 | -0.99 | 0.46 |
| ENSMUSP00000025601 | Lpxn | -0.99 | 0.46 | -0.99 | 0.50 |
| ENSMUSP00000069418 | C4b | -0.99 | 0.63 | -0.91 | 0.79 |
| ENSMUSP00000105211 | Scand1 | 0.98 | 1.50 | 0.97 | 1.43 |
| ENSMUSP00000048078 | Mrpl47 | 0.98 | 1.50 | 0.99 | 1.94 |
| ENSMUSP00000048573 | Agpat1 | -0.98 | 0.76 | -0.97 | 0.80 |
| ENSMUSP00000030296 | Txndc12 | -0.98 | 0.63 | -0.97 | 0.73 |
| ENSMUSP00000092644 | Fam133b | 0.98 | 1.55 | 0.94 | 1.34 |
| ENSMUSP00000080743 | Ptcd3 | 0.98 | 1.57 | 0.95 | 1.33 |
| ENSMUSP00000014421 | Ankrd17 | 0.98 | 1.51 | 0.92 | 1.36 |
| ENSMUSP00000003669 | Homer3 | -0.98 | 0.78 | 0.95 | 1.25 |
| ENSMUSP00000035177 | Mrpl3 | 0.97 | 1.48 | 0.97 | 1.38 |
| ENSMUSP00000034226 | Fam192a | -0.97 | 0.76 | -0.99 | 0.71 |
| ENSMUSP00000118558 | Tia1 | 0.97 | 1.50 | 0.99 | 1.58 |
| ENSMUSP00000115934 | Dhodh | -0.97 | 0.81 | 0.95 | 1.21 |
| ENSMUSP00000083928 | Man2a1 | 0.96 | 1.37 | 0.98 | 1.59 |
| ENSMUSP00000033993 | Gtf2e2 | 0.96 | 1.43 | 0.99 | 1.70 |
| ENSMUSP00000034136 | Gpt2 | -0.96 | 0.68 | -0.96 | 0.68 |
| ENSMUSP00000006854 | Usp19 | -0.96 | 0.81 | -0.98 | 0.77 |
| ENSMUSP00000106234 | Wdr76 | 0.96 | 1.39 | 0.94 | 1.31 |
| ENSMUSP00000112977 | Mrpl1 | 0.95 | 1.37 | 0.99 | 1.62 |
| ENSMUSP00000021913 | Auh | 0.95 | 1.38 | 0.97 | 1.43 |
| ENSMUSP00000097599 | Pitpnm1 | -0.95 | 0.75 | -0.96 | 0.74 |
| ENSMUSP00000112266 | Brms1 | -0.95 | 0.75 | -0.99 | 0.61 |
| ENSMUSP00000067603 | Lcor | 0.95 | 1.42 | 0.96 | 1.40 |
| ENSMUSP00000041912 | Erf | 0.95 | 1.34 | 0.96 | 1.38 |
| ENSMUSP00000049338 | Nipsnap1 | 0.94 | 1.40 | 0.98 | 1.51 |
| ENSMUSP00000028802 | Casc5 | 0.94 | 1.30 | 0.94 | 1.35 |
| ENSMUSP00000109975 | Tprn | 0.93 | 1.32 | 0.98 | 1.49 |
| ENSMUSP00000064084 | Rasal3 | -0.93 | 0.83 | -0.97 | 0.79 |
| ENSMUSP00000039264 | Tap1 | 0.93 | 1.36 | 0.97 | 1.45 |
| ENSMUSP00000105753 | Trmt6 | 0.93 | 1.28 | 0.96 | 1.36 |

**Supplementary Table 8. Phosphopeptides whose expression is altered by the expression of JAK2V617F and JAK2K539L**

Table showing phosphopeptides identified and defined as changing in the two experiments each containing biological replicates for both JAK2V617F and K539L expressing cells. For inclusion in this list a peptide must be seen as ‘changing’ (as defined by the interval in Supplementary Table 1) in both the JAK2V617F and K539L comparisons in at least one experiment and the averaged significance of change must be greater than or equal to 0.9

| **Accession** | **Gene symbol** | **Peptide** | **JAK2V617F:JAK2WT** | | **JAK2K539L:JAK2WT** | |
| --- | --- | --- | --- | --- | --- | --- |
|  |  |  | **Significance of change** | **Ratio** | **Significance of change** | **Ratio** |
| ENSMUSP00000004379 | Emg1 | RFS[Pho]VQEQDWETTPPK | 0.98 | 1.75 | 0.96 | 1.51 |
| ENSMUSP00000101714 | Zmym4 | AAHQES[Pho]DNENEIQIQNQLK | 0.97 | 1.60 | 0.94 | 1.64 |
| ENSMUSP00000033310 | Mki67 | EGHS[Pho]PLSK | 0.94 | 1.61 | 0.95 | 1.68 |
| ENSMUSP00000029699 | Lmna | LRLS[Pho]PSPTSQR | -0.93 | 0.68 | -0.93 | 0.67 |
| ENSMUSP00000033310 | Mki67 | SQS[Pho]PEDLSGVQEVFQTSGHNK | 0.91 | 1.54 | 0.94 | 1.43 |
| ENSMUSP00000021381 | Pnn | RES[Pho]RQESDPEDDDVKKPALQSS[Pho]VVATSK | 0.99 | 2.23 | 0.99 | 1.99 |
| ENSMUSP00000021381 | Pnn | RES[Pho]RQES[Pho]DPEDDDVKKPALQSSVVATSK | 0.99 | 1.98 | 0.98 | 1.71 |
| ENSMUSP00000021381 | Pnn | RESRQES[Pho]DPEDDDVKKPALQSS[Pho]VVATSK | 0.99 | 2.20 | 0.99 | 1.84 |
| ENSMUSP00000027736 | Zc3h11a | RLS[Pho]SASTGKPPLSVEDDFEK | 0.99 | 2.00 | 0.99 | 1.97 |
| ENSMUSP00000029699 | Lmna | LSPSPT[Pho]SQR | -0.99 | 0.50 | -0.99 | 0.48 |
| ENSMUSP00000031841 | Tra2a | AHTPT[Pho]PGIYMGR | 0.99 | 2.62 | 0.99 | 2.75 |
| ENSMUSP00000048383 | Kif4 | TFS[Pho]YDEIHGQDSGAEDSIAK | 0.99 | 2.88 | 0.99 | 2.27 |
| ENSMUSP00000063677 | Plekho2 | SSS[Pho]LGDLLR | -0.99 | 0.51 | -0.98 | 0.62 |
| ENSMUSP00000099487 | Nop56 | KFS[Pho]EEPEVAANFTK | 0.99 | 1.89 | 0.99 | 1.77 |
| ENSMUSP00000101720 | Foxk2 | EGS[Pho]PAPLEPEPGASQPK | 0.99 | 1.91 | 0.99 | 1.80 |
| ENSMUSP00000108103 | Ptpn6 | DLS[Pho]GPDAETLLK | 0.99 | 2.05 | 0.99 | 1.76 |
| ENSMUSP00000122420 | Cdc23 | RVS[Pho]PLNLSSVTP | -0.99 | 0.26 | -0.99 | 0.20 |
| ENSMUSP00000123852 | MYC | KFELLPTPPLS[Pho]PSRR | 0.99 | 2.31 | 0.99 | 2.21 |
| ENSMUSP00000005003 | Lbr | KSGSISSS[Pho]PSR | -0.99 | 0.55 | -0.99 | 0.57 |
| ENSMUSP00000029911 | Sfrs18 | SKFDS[Pho]DEEDEDAENLEAVSSGK | 0.99 | 1.70 | 0.99 | 2.40 |
| ENSMUSP00000061012 | Hmgn1 | KVS[Pho]ADGAAK | 0.99 | 1.75 | 0.98 | 1.74 |
| ENSMUSP00000072092 | Tmpo | GPPDFSS[Pho]DEEREPT[Pho]PVLGSGASVGR | 0.99 | 1.77 | 0.98 | 1.66 |
| ENSMUSP00000081611 | Srcap | HNELGTTTGGS[Pho]PEN[Dea]GEGTELAITPPAVK | 0.99 | 1.72 | 0.96 | 1.50 |
| ENSMUSP00000094515 | Hnrnpul2 | SKS[Pho]PPPPEEEAKDEEEDQTLVNLDTYTSDLHFQISK | -0.99 | 0.55 | -0.97 | 0.61 |
| ENSMUSP00000023226 | Plec | RTSS[Pho]EDNLYLAVLR | -0.98 | 0.60 | -0.97 | 0.62 |
| ENSMUSP00000047792 | Spna2 | KLDPAQSAS[Pho]RENLLEEQGSIALR | -0.98 | 0.58 | -0.98 | 0.57 |
| ENSMUSP00000089948 | Mbd3 | VRYDSS[Pho]NQVK | 0.98 | 1.71 | 0.97 | 1.57 |
| ENSMUSP00000105923 | Elmsan1 | AGT[Pho]FIAP[Oxi]PVYSN[Dea]ITPYQS[Deh]HLRSPVR | 0.98 | 1.83 | 0.99 | 1.79 |
| ENSMUSP00000096547 | Smarca4 | EVDYSDS[Pho]LTEK | 0.97 | 1.68 | 0.97 | 1.56 |
| ENSMUSP00000100546 | Gm11275 | RIS[Pho]GLIYEETR | 0.97 | 1.77 | 0.98 | 1.45 |
| ENSMUSP00000112104 | Hnrnpk | DYDDMS[Pho]PR | 0.97 | 1.69 | 0.96 | 1.48 |
| ENSMUSP00000018909 | Fxr2 | TDGS[Pho]IS[Pho]GDRQPVTVADYISR | 0.97 | 1.60 | 0.97 | 1.55 |
| ENSMUSP00000101015 | Arid3a | AAAAGLGHPSS[Pho]PGGSEDGPPISGDEDTAR | -0.97 | 0.66 | -0.99 | 0.58 |
| ENSMUSP00000032997 | Lat | ELPVSYDSTS[Pho]TESLYPR | -0.96 | 0.64 | -0.98 | 0.61 |
| ENSMUSP00000031841 | Tra2a | AHT[Pho]PTPGIY | 0.96 | 1.61 | 0.97 | 1.56 |
| ENSMUSP00000005003 | Lbr | KSGS[Pho]ISSSPSR | -0.95 | 0.69 | -0.96 | 0.66 |
| ENSMUSP00000027736 | Zc3h11a | RLSS[Pho]ASTGKPPLSVEDDFEK | 0.95 | 1.71 | 0.99 | 1.92 |
| ENSMUSP00000032949 | Coro1a | RAT[Pho]PEPSGTPSSDTVSR | -0.95 | 0.64 | -0.97 | 0.72 |

**Excel Spread sheets**

**Supplementary Table 3 – All Proteins identified in the SILAC labelled cells**

Table showing all the 5021 proteins identified and quantified in whole cell lysates from JAK2 Wild Type and JAK2 V617F expressing cells labelled with SILAC. The error factor represents the coefficient of variability over all the redundant quantifiable peptides. It is calculated as the standard deviation of the naturally logarithmized ratios times 100

**Supplementary Table 5 – All nuclear proteins identified in the iTRAQ labelled nuclear extracts**

Table showing all 3391 nuclear proteins identified across three biological replicate experiments and one instrument technical replicate of Ba/F3 cells transfected with JAK2 V617F and JAK2 K539L. The first four columns display protein and gene accessions, gene symbol and name. The next twelve columns give information from the search results for the identification of the protein in each experiment (blanks if not identified). ‘’Coverage’’ indicates what percentage of the protein sequence was identified. ‘’Unique peptides’’ indicates the number of unique peptides discovered whilst ‘’Peptides used’’ indicates the number of unique peptides used to derive the quantification. The remaining columns display quantification ratios (against control) and associated p-values and error factors for each replicate. The data includes 3 biological replicates and one instrumentation technical replicate. Data consists of 53,336 peptides with 27,895 having associated quantification.

**Supplementary Table 7 – All Phosphopeptides identified in the iTRAQ labelled nuclear extracts.**

Table showing all nuclear phospho peptides identified (2238) with associated quantification (2090) information across the two experiments with biological replicates in each experiments. The first three columns give protein and gene accessions, gene symbol and name of the protein from which each peptide originates. The next two columns list the modification assigned to each peptide and the peptide sequence. The following four columns detail the confidence in the peptide identification and number of spectra used to generate the quantification data in each run. Then, for each JAK2 V617F and JAK2 K539L versus JAK2 WT comparison, the number of runs where a value for the quantification ratio was returned, the calculated significance of change and the ratio and significance of change for each run is given. The last six columns, ‘rep’, display the average values across the runs
